# Benefits of Image-Free Robotic-Assisted Total Knee Arthroplasty in Early Postoperative Recovery and Tibial Alignment Accuracy

**DOI:** 10.7759/cureus.92044

**Published:** 2025-09-11

**Authors:** Kazu Matsumoto, Daichi Ishimaru, Kazuki Sohmiya, Nobuo Terabayashi

**Affiliations:** 1 Department of Orthopedics, Gifu Seiryu Hospital, Gifu, JPN; 2 Department of Orthopedics, Gifu University Hospital, Gifu, JPN

**Keywords:** conventional, muscle strength, patient recovery, range of motion, robot, robotic-assisted total knee arthroplasty, total knee arthroplasty

## Abstract

Background

Robotic-assisted total knee arthroplasty (RA-TKA) has been introduced to improve implant alignment and soft tissue balance, potentially enhancing early functional recovery and patient satisfaction. The Navio system (Smith & Nephew, Inc., Memphis, TN, USA) is an imageless, handheld, semi-active robotic platform, but evidence regarding its short-term outcomes remains limited. This study aimed to compare early postoperative recovery, pain, rescue analgesic use, and implant alignment between Navio RA-TKA and conventional TKA.

Methods

We retrospectively reviewed 113 knees in 100 patients undergoing primary bi-cruciate stabilized TKA for varus osteoarthritis between April 2019 and July 2022. Conventional TKA (55 knees) was performed using extramedullary guides, and Navio RA-TKA (58 knees) utilized haptic-guided burr resection. Postoperative quadriceps muscle strength (MS), knee range of motion (ROM), visual analogue scale (VAS) pain scores, femoral circumference, rescue analgesic doses, and coronal component alignment were assessed up to postoperative day (POD) 14.

Results

Quadriceps MS recovery was significantly faster in the RA-TKA group at POD three, seven, 10, and 14 (all p<0.05). ROM recovery was greater on POD 10 (p=0.0231). Rescue analgesic use was lower with RA-TKA (4.12±7.26 vs. 7.98±12.1 doses, p=0.0399). No significant differences were found in VAS scores or the femoral circumference. Tibial component alignment within 2° of the mechanical axis was achieved more frequently in the RA-TKA group (96.4% vs. 72.2%, p=0.0004).

Conclusion

Navio RA-TKA resulted in faster early functional recovery, reduced analgesic requirements, and improved tibial alignment accuracy compared with conventional TKA. These benefits may enhance early patient satisfaction and support the broader adoption of imageless, handheld robotic systems. Prospective randomized studies with long-term follow-up are warranted.

## Introduction

Patient satisfaction remains a crucial outcome measure in total knee arthroplasty (TKA), with reported satisfaction rates ranging from 80% to 90% [1]. Persistent dissatisfaction has been attributed to factors such as lower implant survivorship, suboptimal functional recovery, and the need for revision surgery, which are often associated with component malalignment and soft tissue imbalance [2-6]. However, comprehensive reviews indicate that while component malalignment and soft tissue imbalance are known contributors to patient dissatisfaction following TKA, they represent only a part of a much broader array of factors influencing outcomes [7]. To address these issues, robotic-assisted TKA (RA-TKA) has recently been introduced as a potential solution to improve the accuracy of implant positioning and soft tissue balance.

Robotic systems for TKA can be broadly classified into passive, semi-active, and active systems [8]. Semi-active robotic systems allow the surgeon to retain control over bone resection and implant positioning while benefiting from enhanced precision. Among these, image-dependent and imageless systems represent two distinct approaches. The Mako Robotic Arm Interactive Orthopaedic System (Stryker Ltd, Kalamazoo, MI, USA) is a well-established, image-guided semi-active system. In contrast, the Navio Surgical System (Smith & Nephew, Inc., Memphis, TN, USA) is an imageless, handheld robotic sculpting system introduced for TKA in 2017 [9].

Several studies have demonstrated that RA-TKA achieves more accurate implant alignment than conventional techniques [10-12]. Nevertheless, other reports have found no significant improvement in functional outcomes or patient satisfaction with robotic TKA compared to conventional TKA [13,14]. Hampp et al. [10] reported superior accuracy of component positioning with Mako RA-TKA compared to manual TKA in a cadaveric study. Similarly, Bollars et al. [11] observed significantly fewer alignment deviations with the Navio system compared to conventional TKA. Accurate implant alignment is expected to translate into improved functional outcomes.

Early functional recovery is another critical determinant of patient satisfaction. Kayani et al. [15] demonstrated that Mako RA-TKA facilitated reduced postoperative pain and accelerated early functional recovery compared with conventional TKA. Additional studies have reported short-term functional improvements with the Mako system, as assessed by the Knee Society Score and the Western Ontario and McMaster Universities Osteoarthritis Index [16,17]. However, limited evidence is available regarding early postoperative outcomes, particularly within the first 14 days, following Navio RA-TKA.

Therefore, this study aimed to compare early postoperative outcomes, including functional recovery, pain intensity, rescue analgesic use, and implant alignment, between Navio RA-TKA and conventional TKA. We hypothesised that Navio RA-TKA would provide superior early functional recovery and improved implant alignment compared with conventional TKA.

## Materials and methods

Participants

**Figure 1:**
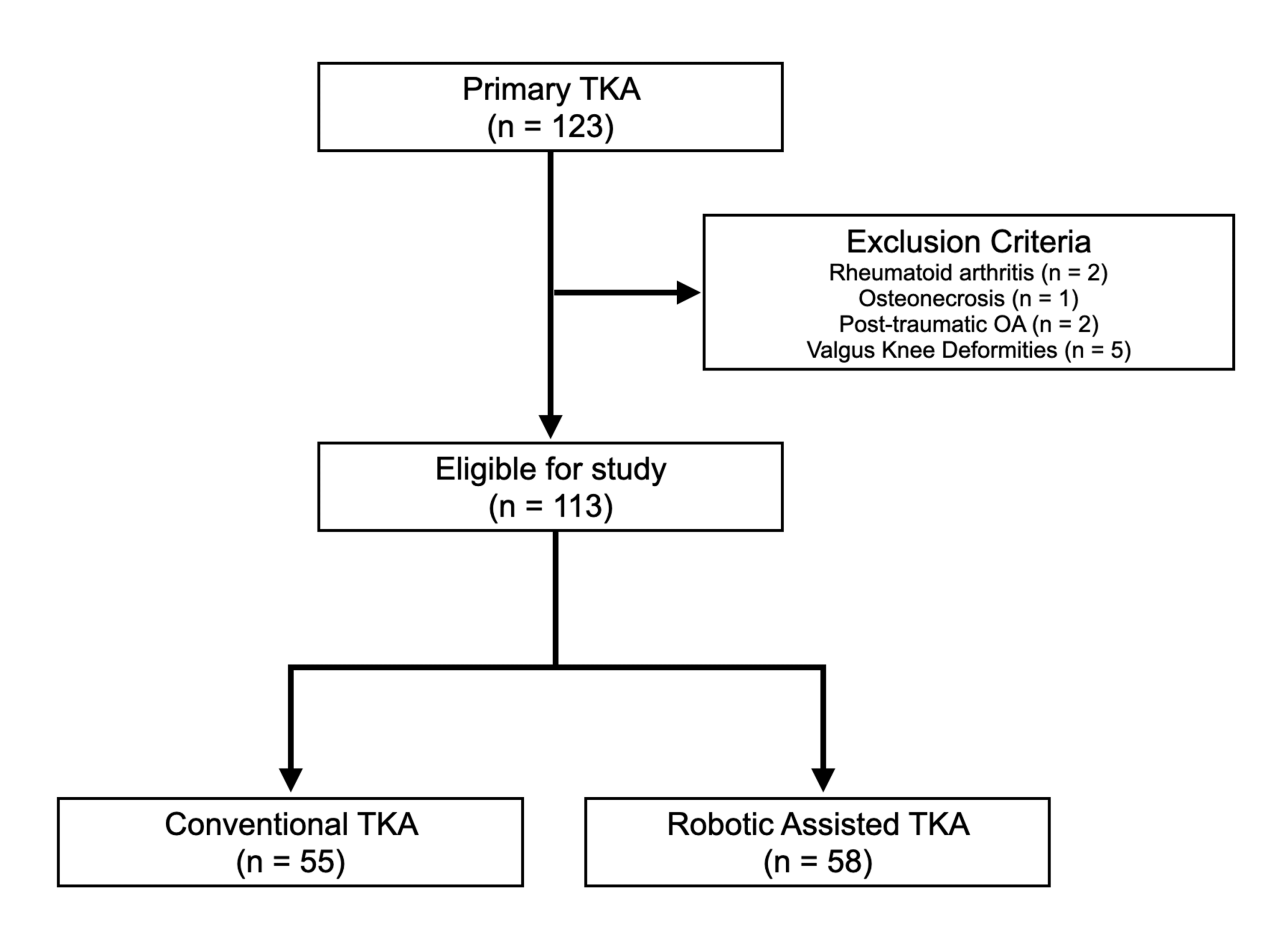
Study flow diagram Flowchart illustrating the study design. Patients with primary osteoarthritis who met the inclusion criteria were allocated to either conventional total knee arthroplasty (TKA) or Navio robotic-assisted TKA (RA-TKA).

Between April 2019 and July 2022, a total of 123 primary bi-cruciate stabilized (BCS) TKAs (Journey II; Smith & Nephew, Tokyo, Japan) were performed in 110 patients at our institution. Patients with primary osteoarthritis (OA) and varus knee deformities were included. Exclusion criteria comprised rheumatoid arthritis, osteonecrosis of the knee, post-traumatic OA, and valgus knee deformities. After applying the inclusion and exclusion criteria, 113 knees in 100 patients were eligible for analysis (Figure 1).

Conventional TKA was performed in 55 knees (51 patients) between April 2019 and March 2021, whereas Navio RA-TKA was performed in 58 knees (49 patients) between April 2021 and July 2022 (Figure 1). Patients were divided into two groups: the control group (conventional TKA) and the RA-TKA group (Navio). All procedures in both groups were performed by the same senior surgical team, led by two experienced knee arthroplasty surgeons, to minimize variability in surgical technique. No changes in the core surgical team occurred during the study period. 

The Gifu Seiryu Hospital Review Board granted approval for the study (approval number: #071).

Surgical procedures

Conventional TKA (Control Group)

Extramedullary (EM)-guided TKA was performed according to established protocols [18,19]. The procedure involved a medial parapatellar approach with resection of the anterior and posterior cruciate ligaments. Tibial and femoral bone cuts were performed using EM guides, with alignment referenced to the Whiteside’s line and the mechanical axis. The femoral component was aligned at 90° to the mechanical axis in the coronal plane and 3° of flexion in the sagittal plane to avoid anterior cortex notching. Femoral rotation was determined via gap balancing, and tibial component rotation was determined using the range-of-motion (ROM) technique [20]. All components were cemented.

Robotic-Assisted TKA (RA-TKA Group)

Navio RA-TKA followed the same approach until joint exposure. Two tracking pins were placed in the proximal tibia and distal femur. Anatomical landmarks, including the hip center, were registered using a probe to create a virtual three-dimensional knee model. Dynamic soft tissue balancing was performed across the full ROM under varus and valgus stress. We note that such intraoperative balancing with robotic assistance is not yet a universally performed or routinely adopted technique, and practices may vary between institutions. The implant size, position, and insert thickness were adjusted to optimize soft tissue balance and alignment. Bone cuts were performed using a high-speed 5 mm burr, with haptic feedback guiding the burr within predefined cutting boundaries. Tibial saw guide pinholes were prepared using a 5 mm burr. After the trial component placement, final balancing and gap assessment were confirmed before cementing the components.

Postoperative rehabilitation protocol

Postoperative rehabilitation was initiated on postoperative day (POD) one in all patients, under the supervision of the same group of physiotherapists. The protocol included early mobilization, active and passive ROM exercises, and progressive quadriceps strengthening. The frequency, duration, and type of exercises were standardized for both groups. The outcome measurements are given below.

Pain and Functional Recovery

Postoperative pain was assessed using a 100-mm visual analogue scale (VAS) at rest (rVAS) and during movement (mVAS) preoperatively and on POD three, seven, 10, and 14 [21]. Functional recovery was evaluated by quadriceps muscle strength (MS) and knee ROM. Quadriceps MS was measured using a handheld dynamometer (Isoforce, OG GIKEN Co., Ltd., Okayama, Japan), and knee ROM was measured with a goniometer following established procedures [22]. Manual MS testing, as described by Kendall et al., provided the methodological basis for quadriceps evaluation [23]. Preoperative MS and ROM were defined as 100%, and postoperative recovery was expressed as the percentage of preoperative values.

Soft Tissue Swelling

Femoral circumference was measured 10 cm proximal to the superior pole of the patella to evaluate soft tissue swelling. Preoperative circumference was considered 100%, and postoperative changes were expressed as a percentage of preoperative values.

Rescue Analgesia

All patients received oral celecoxib (200 mg every eight hours) for 14 days postoperatively. In addition to the standardized celecoxib regimen, no regional anesthesia (e.g., femoral or adductor canal nerve block) or local infiltration analgesia was performed in either group. Compliance with the celecoxib regimen exceeded 95% in both groups. Additional analgesia (intravenous or oral acetaminophen) was administered as needed, and the total number of rescue doses up to POD 14 was recorded.

Radiographic Evaluation

Preoperative and postoperative anteroposterior full-length lower limb radiographs were obtained to measure the femorotibial angle (FTA) and coronal alignment of femoral (α) and tibial (β) components [18,19]. All radiographic measurements were performed independently by two blinded observers. Inter- and intra-observer reliability were assessed in a random subset of 20 cases, with intraclass correlation coefficients (ICCs) exceeding 0.85 for all parameters. Proper alignment was defined as α or β deviation <2° from the mechanical axis.

Statistical Analysis

The primary outcome was recovery of quadriceps muscle strength (MS) within 14 PODs. An a priori power analysis determined that a minimum of 28 patients per group was required [24]. Continuous variables were presented as mean ± standard deviation and were compared using either the Student’s t-test or the Wilcoxon rank-sum test, depending on data distribution. Categorical variables were compared using the Chi-square test or Fisher’s exact test, as appropriate. All statistical analyses were performed using GraphPad Prism (version 5.0; GraphPad Software, San Diego, CA, USA), with statistical significance defined as p<0.05.

## Results

Demographic and clinical characteristics

Baseline demographics, including age, sex, body mass index (BMI), and preoperative FTA, were comparable between groups (Table 1). 

**Table 1:** Baseline demographic and clinical characteristics of patients undergoing conventional TKA and Navio RA-TKA TKA: Total knee arthroplasty; RA-TKA: Robotic-assisted Total knee arthroplasty. No significant differences were observed between groups in age, sex, body mass index (BMI), or preoperative femorotibial angle (FTA). For continuous variables (age, height, body weight, BMI, and FTA), we assessed normality and performed comparisons using the Student’s t-test. For the categorical variable (Sex), we used the Chi-square test.

	Control group (N=54)	RA-TKA group (N=56)	Test statistics	P value
Age (years)	73.4 ± 7.8	72.6 ± 9.6	t = 0.12	0.9063
Sex (F:M)	40:15	44:14	χ² = 0.14	0.7053
Height (m)	1.53 ± 0.096	1.52 ± 0.096	t = 0.08	0.9364
Body weight (kg)	62.9 ± 13.9	60.7 ± 9.6	t = 0.76	0.4496
BMI	26.8 ± 4.4	26.3 ± 4.3	t = 0.69	0.4944
FTA (°)	184.2 ± 12.9	185.0 ± 8.9	t = 0.69	0.4922

Postoperative pain

**Figure 2:**
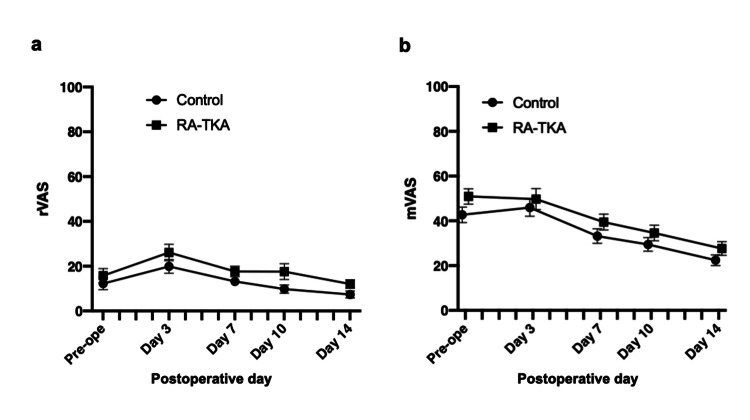
Postoperative pain scores following TKA TKA: Total knee arthroplasty; RA-TKA: Robotic-assisted Total knee arthroplasty. (a) Visual analogue scale at rest (rVAS) and (b) Visual analogue scale (mVAS) at preoperative baseline and postoperative days (PODs) 3, 7, 10, and 14. No significant differences were observed between groups at any time point. Pain was assessed using a 100-mm VAS [21].

No significant differences were observed in mean rVAS or mVAS scores between groups across all time points (Figures 2A-2B). 

Functional recovery

**Figure 3:**
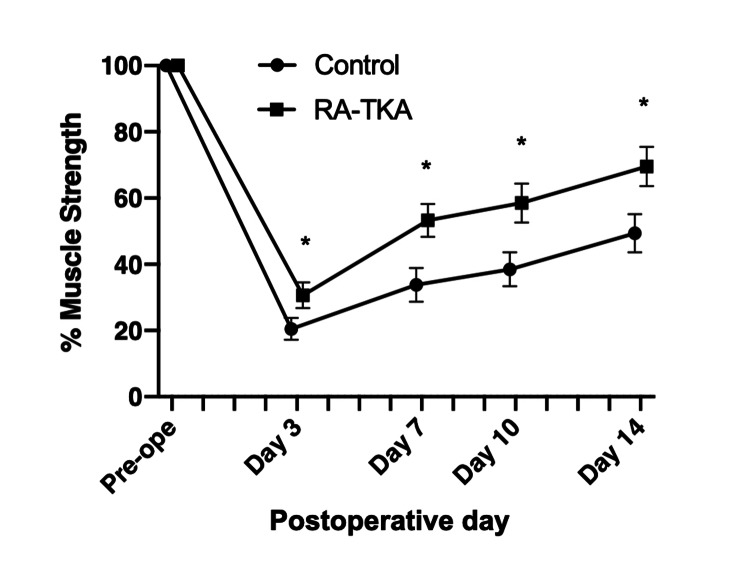
Quadriceps muscle strength (MS) recovery expressed as a percentage of preoperative values at PODs 3, 7, 10, and 14 TKA: Total knee arthroplasty; RA-TKA: Robotic-assisted Total knee arthroplasty; POD: Postoperative day. The RA-TKA group demonstrated significantly faster recovery than the conventional TKA group at all time points (p<0.05). MS was measured using a handheld dynamometer (Isoforce, OG GIKEN Co., Ltd., Okayama, Japan), based on the methodology described by Kendall et al. [23].

Quadriceps MS recovery was significantly faster in the RA-TKA group than in the control group on POD three (p=0.0266), POD seven (p=0.0104), POD 10 (p=0.0384), and POD 14 (p=0.0068) (Figure 3). 

**Figure 4:**
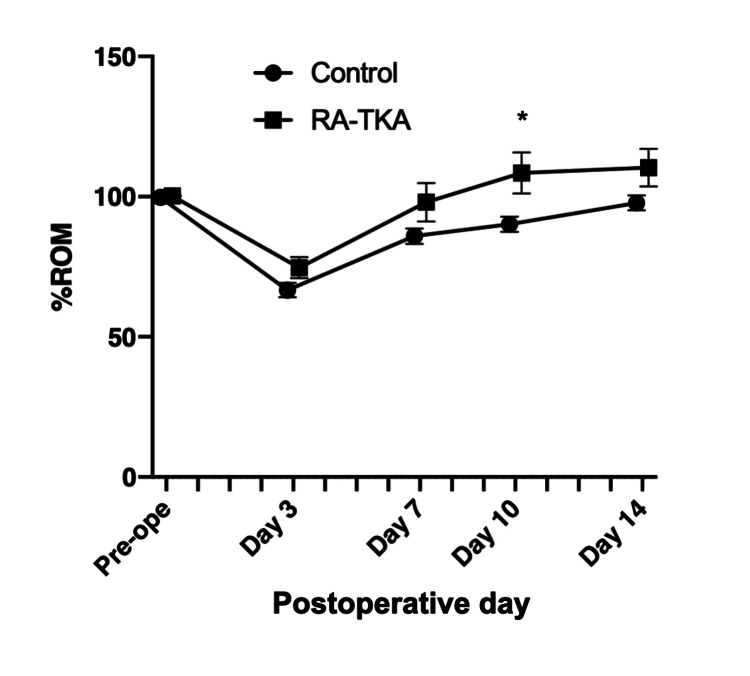
Knee range of motion (ROM) recovery expressed as a percentage of preoperative values at PODs 3, 7, 10, and 14 TKA: Total knee arthroplasty; RA-TKA: Robotic-assisted Total knee arthroplasty; POD: Postoperative day. The RA-TKA group showed significantly greater ROM recovery on POD 10 (p<0.05). ROM was measured using a goniometer according to established procedures [22].

Knee ROM recovery was greater in the RA-TKA group, achieving significance on POD 10 (p=0.0231) but not at other time points (Figure 4). 

Soft tissue swelling

**Figure 5:**
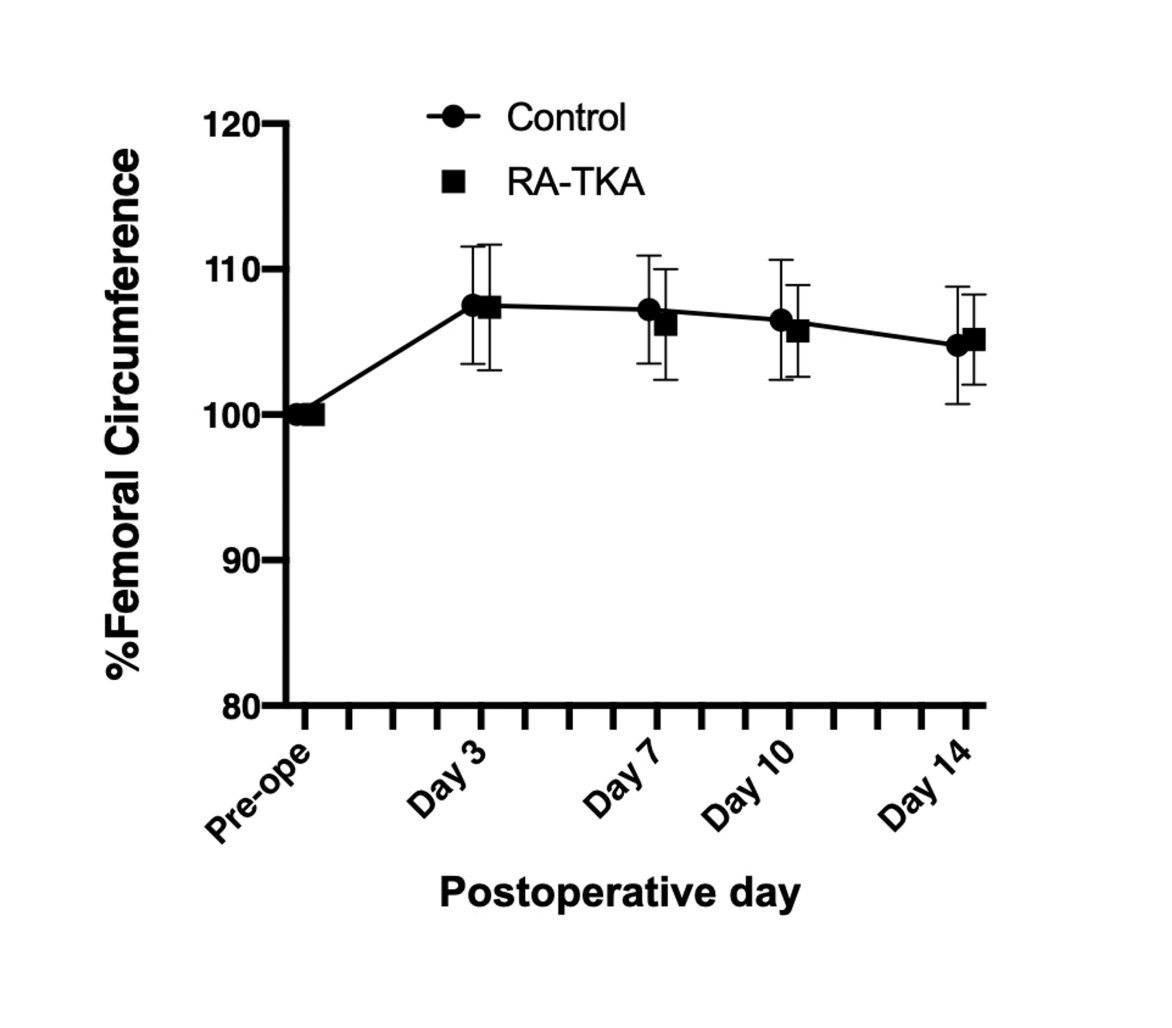
Changes in femoral circumference (soft tissue swelling) expressed as a percentage of preoperative values TKA: Total knee arthroplasty; RA-TKA: Robotic-assisted Total knee arthroplasty; No significant differences were found between groups throughout the postoperative period.

No significant differences were observed in femoral circumference changes between groups throughout the postoperative period (Figure 5). 

Rescue analgesia

The RA-TKA group required significantly fewer rescue doses of analgesics compared to the control group (4.12±7.26 vs. 7.98±12.1, p=0.0399).

Radiographic outcomes

**Figure 6:**
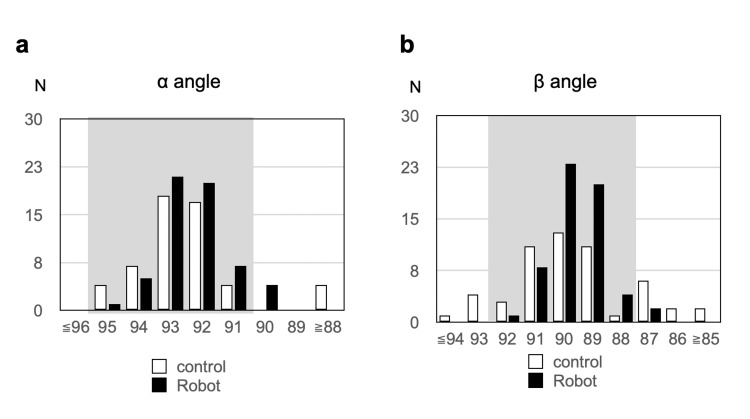
Representative postoperative coronal radiographs of component alignment Control group: Total knee arthroplasty; Robot group: Robotic-assisted Total knee arthroplasty. (a) Distribution of femoral α angles (coronal alignment) in the two groups. The grey area indicates less than 2° of deviation from the mechanical axis in the coronal plane. (b) Distribution of tibial β angles (coronal alignment) in the two groups. The grey area indicates less than 2° of deviation from the mechanical axis in the coronal plane

The femoral component coronal alignment (α angle) was comparable between groups (92.2°±2.1° vs. 92.3°±1.1°, p=0.4232). Proper femoral alignment (α deviation <2°) was achieved in 92.6% of control and 92.9% of RA-TKA knees (p=0.9579). Tibial component alignment (β angle) was similar in mean values (89.6°±2.2° vs. 89.6°±1.0°, p=0.4427), but proper alignment (β deviation <2°) was achieved significantly more frequently in the RA-TKA group (96.4% vs. 72.2%, p=0.0004) (Table 2, Figure 6). 

**Table 2:** Postoperative coronal alignment of femoral (α) and tibial (β) components in conventional TKA and Navio RA-TKA TKA: Total knee arthroplasty; RA-TKA: Robotic-assisted Total knee arthroplasty. Proper alignment was defined as α or β deviation <2° from the mechanical axis. The RA-TKA group achieved significantly higher rates of proper tibial alignment compared with the control or conventional TKA group (p=0.0004). Student’s t-tests were used for continuous variables (α and β angles), and Chi-square tests were used for categorical variables (percentage within 2°). The test statistics (t and χ² values) are now reported alongside the p-values.

	Control group (N=54)	RA-TKA group (N=56)	Test statistics	Pvalue
Coronal alignment of femoral component (α angle, °)	92.2 ± 2.1°	92.3 ± 1.1°	t = 0.80	0.4232
Coronal alignment of tibial component (β angle, °)	89.6 ± 2.2°	89.6 ± 1.0°	t = 0.77	0.4427
Femoral component varus/valgus: % within 2°	92.6%	92.9%	χ² = 0.00	0.9579
Tibial component varus/valgus: % within 2°	72.2%	96.4%	χ² = 12.5	0.0004

## Discussion

The key finding of this study is that image-free, handheld RA-TKA (Navio) significantly improved early functional recovery, including quadriceps MS and ROM, and reduced analgesic requirements compared with conventional TKA. Moreover, RA-TKA achieved superior tibial component alignment accuracy.

The improved accuracy of component positioning with RA-TKA has been consistently reported [10-12,25]. Our findings are consistent with Bollars et al. [11], who reported fewer alignment deviations with Navio RA-TKA than conventional TKA, and with Horteur et al. [23], who demonstrated superior tibial rotational alignment using 3D-CT. However, other investigators have reported no significant differences in clinical outcomes or patient satisfaction between robotic and conventional TKA, despite improved alignment [13,14]. The enhanced alignment accuracy likely contributes to the improved early functional recovery observed in our study.

Although previous studies demonstrated reduced postoperative pain with Mako RA-TKA [15,26], we found no significant differences in VAS scores between groups. However, the RA-TKA group required fewer rescue analgesic doses, suggesting a potential reduction in overall pain burden. This discrepancy may reflect differences in analgesic protocols or the subjective nature of pain assessment.

Quadriceps MS recovery was significantly better in the RA-TKA group, aligning with findings by Kayani et al. [15], who reported faster achievement of straight-leg raise following Mako RA-TKA. To our knowledge, this is the first study to directly evaluate quadriceps strength in Navio RA-TKA. The faster recovery may be attributable to less soft tissue trauma, as robotic haptic boundaries minimize unnecessary bone resection and periarticular soft tissue release [27,28]. Although femoral circumference did not differ significantly between groups, subtle differences in soft tissue handling may still have contributed to the improved recovery.

The early ROM recovery in the RA-TKA group further supports the hypothesis that reduced soft tissue trauma facilitates faster functional rehabilitation. While the early benefits may not persist long-term, as shown in previous long-term studies [29,30], the early recovery advantage could enhance patient satisfaction and facilitate earlier return to daily activities.

This study has several limitations. First, it was retrospective, and the two groups were operated in different time periods, which may have introduced temporal bias. Changes in perioperative management strategies, rehabilitation protocols, and institutional practices over time could have influenced the outcomes independently of the surgical technique. Second, the follow-up was limited to 14 PODs; thus, long-term functional outcomes and implant survivorship were not evaluated. Third, implant alignment was assessed using plain radiographs, which are less precise than CT-based measurements, although inter- and intra-observer reliability was high. Furthermore, it should be emphasized that comprehensive reviews indicate that while component malalignment and soft tissue imbalance are known contributors to patient dissatisfaction following TKA, they represent only a part of a much broader array of factors influencing outcomes. Finally, all Navio RA-TKA procedures in this study were performed during the initial adoption phase at our institution. Although the learning curve may have influenced surgical efficiency, previous literature suggests that alignment accuracy is maintained even in early cases.

## Conclusions

This study demonstrated that Navio RA-TKA resulted in significantly faster early functional recovery, as evidenced by improved quadriceps strength and knee ROM, compared with conventional TKA. In addition, patients who underwent robotic-assisted procedures required fewer rescue analgesics, indicating a reduced postoperative pain burden. The Navio RA-TKA system also achieved superior tibial component alignment accuracy, supporting its role in enhancing surgical precision.

Although pain scores and soft tissue swelling did not differ significantly between groups, the observed improvements in early functional outcomes suggest that Navio RA-TKA may contribute to greater patient satisfaction and an earlier return to daily activities. These findings highlight the potential clinical benefits of robotic assistance in TKA, warranting further prospective randomized trials with longer follow-up to confirm their impact on long-term function and implant survivorship.
